# Development of a Recombinant Cell-Based Indirect Immunofluorescence Assay for the Determination of Autoantibodies against Soluble Liver Antigen in Autoimmune Hepatitis

**DOI:** 10.1155/2013/572815

**Published:** 2013-01-16

**Authors:** Christiane Radzimski, Christian Probst, Bianca Teegen, Kristin Rentzsch, Inga Madeleine Blöcker, Cornelia Dähnrich, Wolfgang Schlumberger, Winfried Stöcker, Dimitrios P. Bogdanos, Lars Komorowski

**Affiliations:** ^1^Institute of Experimental Immunology, Euroimmun AG, Seekamp 31, 23560 Lübeck, Germany; ^2^Institute of Liver Studies, School of Medicine, King's College London, Denmark Hill, London SE5 9RS, UK

## Abstract

Autoantibodies against soluble liver antigen (SLA) are specific markers for autoimmune hepatitis (AIH) type 1. In contrast to the determination of other AIH-associated autoantibodies by indirect immunofluorescence assay (IFA), detection of anti-SLA relied up to now on ELISA or immunoblot based on bacterially expressed recombinant protein. In order to develop a complementary IFA substrate, SLA isoform 1 was recombinantly produced in the human cell line HEK293 and controlled by a rabbit hyperimmune serum against SLA. The recombinant cells were used in IFA (RC-IFA) to analyze sera from 20 AIH patients with anti-SLA positivity predetermined by ELISA together with 80 controls (20 anti-SLA negative AIH, 15 primary biliary cirrhosis, 15 HCV, and 30 healthy blood donors). Using RC-IFA, anti-SLA was detected in all ELISA positive AIH sera but in none of the controls. Furthermore, a cytosolic fraction of HEK293 containing SLA was able to neutralize the autoantibodies in all positive sera in a dose-dependent manner. HEK293 cells expressing SLA are a valid substrate for the serodiagnosis of AIH relevant autoantibodies by IFA. In concert with cryosections of primate liver, rat kidney, rat liver, rat stomach, and HEp-2 cells, they enable the parallel determination of all autoantibodies associated with autoimmune liver diseases.

## 1. Introduction

Autoimmune hepatitis (AIH) is a serious chronic liver disease that affects both sexes and all ages and races [1]. Accurate diagnosis allows for early administration of immunosuppressive treatment which sharply decreases the disease activity in the great majority of the cases [2–4].

The clinical diagnosis of AIH is challenging at times, such that autoantibody markers are commonly used to assist clinicians in their decision [2, 5]. Antinuclear antibodies (ANA) and smooth muscle autoantibodies (SMA) define type 1 AIH (AIH-1), and anti-liver kidney microsomal type 1 (anti-LKM1) and anti-liver cytosol type 1 (anti-LC1) characterize type 2 (AIH-2). Additionally, antibodies against soluble liver antigen (SLA) were found to be highly specific markers of AIH [6–9]. All of these autoantibodies are routinely tested in the differential diagnosis of autoimmune liver diseases in general and AIH in particular [2, 5, 9].

Testing of anti-SLA is included in the recommendations of the International Autoimmune Hepatitis Group (IAIHG) [10]: the early revised criteria that were issued mainly for the better definition of AIH cases involved in research studies [11, 12] and the most recent simplified criteria for the diagnosis of AIH issued to help clinicians in the routine clinical practice [13]. Despite its limited clinical sensitivity of 7–19% [14–16], dependent on the ethnical background as it has been estimated by commercially available test systems, the testing for anti-SLA antibodies seems to be justified because they can be considered pathognomonic markers of AIH, with specificity close to 100% [14].

Finally, both the IAIHG and the American Association for the Study of Liver Diseases (AASLD) guidelines advice routine testing of anti-SLA [2, 12, 13], as these autoantibodies appear to be the only ones for which a consensus has been reached regarding their clinical relevance and in particular their ability to identify AIH patients with more severe disease and worse outcome [17–23].

With the exception of anti-SLA, AIH-associated autoantibodies are routinely detected by indirect immunofluorescence assay (IFA)—still the gold—standard for the detection of ANA and SMA, with or without complementary enzyme-linked immunosorbent assays (ELISA) or line/dot blots based on the target antigens of the respective autoantibodies [5, 24]. For IFA, rodent liver, kidney and stomach tissues, and HEp-2 cells are in general used as test substrates. In contrast, widely available assays for anti-SLA have so far relied on bacterially expressed recombinant protein employed in ELISA or blot assays.

The current diagnostic approaches suffer from two severe limitations: first, there is no uniform assay platform such that laboratories either need to introduce an additional test for anti-SLA next to IFA or have to employ less competent ELISA and blot assays for ANA and SMA to avoid IFA, and second the nowadays available anti-SLA assays are only able to detect antibody reactivity to primary structures whilst missing responses against conformational epitopes. But comprehensive epitope mapping analyses demonstrated that SLA epitopes are mainly conformational [21]. Therefore, eukaryotically expressed SLA was proposed as a more competent target antigen due to the alleged presence of conformational epitopes that are not presented by the bacterially expressed antigen [6].

In the present study, an IFA based on eukaryotically overexpressed SLA was developed in order to complement the portfolio of commonly use substrates for the testing of autoantibodies associated with autoimmune liver diseases.

## 2. Material and Methods

### 2.1. Serum Samples

As positive controls, 20 serum samples containing anti-SLA, determined with commercially available ELISA and line blot (Euroimmun, Germany) based on the SLA variant first described by Wies et al. [6, 14]. The negative control group consisted of 20 patients with autoimmune hepatitis (AIH) type 1 before onset of treatment in which in the conventional anti-SLA antibodies were not detectable. AIH type 1 was defined using the criteria of the 2004 consensus statement of the international AIH group [12]. As further controls, sera from 15 patients with primary biliary cirrhosis and high-titre antimitochondrial antibodies, 15 patients with hepatitis C virus infection, and 30 healthy blood donors without any features suggestive of liver disease were used. In adherence to the Helsinki principles, informed consent from all patients was obtained whose material was used in this study.

#### 2.1.1. Cloning, Expression of SLA in *E. coli*, and Purification

The coding DNA for SLA isoform 1 (Swiss-Prot acc. no. Q9HD40) was obtained by PCR on a cDNA (BioSource, Germany, Genbank acc. no. BX648976), with primers as in Table 1 introducing BsmBI restriction sites. PCR reaction and subsequent ligation with NcoI/XhoI digested pET24d were carried out as in Sitaru et al. [25] thereby adding a C-terminal hexahistidine-tag to the recombinant protein (SLA-His-coli). The final construct was verified by DNA sequencing (MWG Biotech).

SLA-His-coli was expressed in *E. coli Rosetta* (DE3) pLacI (Novagen) and purified under denaturing conditions by immobilized metal chelate affinity chromatography (IMAC) and cation exchange chromatography following the protocol as in Probst et al. [26].

#### 2.1.2. Hyperimmune Serum against SLA

For the generation of a polyclonal serum, rabbits were immunized with SLA-His-coli following a standard 87-day programme (Eurogentec, Belgium). Two normal New Zealand white rabbits were each immunised subcutaneously with 200 *μ*g of recombinant human SLA emulsified in Freund's complete adjuvant (FCA). The animals were boosted with 100 *μ*g of antigen at days 14, 28, and 42 without FCA. At the end of the immunisation protocol, test bleedings were obtained and sera were separated by centrifugation and stored in aliquots at −20°C until used. Pre-immune rabbit sera were used as negative controls. Serum reactivity was controlled by westernblot using SLA-His-coli.

#### 2.1.3. Cloning and Expression of SLA in HEK293

The coding DNA for SLA isoform 1 (Swiss-Prot acc. #Q9HD40) was obtained by PCR on a cDNA (BioSource, Germany, Genbank acc. #BX648976), with primers as in Table 1 introducing BsmBI restriction sites. The PCR reaction was carried out as in Sitaru et al. [25]. After digestion with BsmBI, the amplification product was ligated with NcoI/XhoI-linearized pTriEx-1 (Merck Biosciences, Germany). SLA without a tag was expressed in the human cell line HEK293 (SLA-HEK) after ExGen500-mediated transfection (Fermentas, Germany) according to the manufacturer's instructions. For the preparation of substrates for the indirect immunofluorescence test, HEK293 were grown on sterile glass slides, transfected, and allowed to express the recombinant protein for 48 hours. Slides were washed with PBS, fixed either with acetone or with 1% (w/v) formalin in acetone for 10 minutes at room temperature, air-dried, and stored at −20°C until use.

Alternatively, cells were transfected in standard T-flasks and harvested after 72 hours expression by removing the cell culture medium, scraping the cells off in PBS, washing three times in 20 mmol/L tris-HCl pH 7.4, 150 mmol/L sodium chloride, 5 mmol/L EDTA, 1 mmol/L PMSF and final resuspension in 10 *μ*L/cm^2^ culture surface 20 mmol/L tris-HCl pH 7.4, 10% (w/v) sucrose, 5 mmol/L EDTA, 1 mmol/L PMSF. Cells were frozen at −80°C until further use. For the preparation of cell-free supernatants, the cells were thawed and diluted with 4 volumes of 20 mmol/L tris-HCl pH 7.4, 50 mmol/L potassium chloride, 5 mmol/L EDTA, and 1 mmol/L PMSF. Cell lysis was promoted by dounce homogenization. Cell nuclei were removed by centrifugation at 700 ×g, 4°C for 10 minutes. The supernatant was saved whereas the sediment was lysed for a second time. Finally, the combined supernatants were centrifuged at 100.000 ×g, 4°C for 60 minutes and the resulting supernatant was stored in aliquots at −80°C until further use.

### 2.2. Indirect Immunofluorescence Assay (IFA)

IFA was conducted using slides with four types of substrates: HEK293-SLA and wild-type HEK293, each acetone and formalin fixed, according to the standard instructions for HEp-2 cells (Euroimmun). In some cases, rabbit hyperimmune sera were used in the first step of IFA followed by incubation with Cy3 anti-rabbit IgG (Jackson Research, United Kingdom). In all cases, the incubated slides were evaluated independently by two experts. In neutralization experiments, samples with antigen content were mixed with diluted sera 30 minutes prior to the IFA as described elsewhere [27].

### 2.3. ELISA for the Detection of Human Autoantibodies Against SLA

Microtiter plates (Nunc, Germany) were coated with SLA-His-coli (up to 10 *μ*g/mL) in PBS for 2 hours at 25°C, washed three times with washing buffer (0.05% (w/v) Tween-20 in PBS), and blocked with blocking buffer (0.1% (w/v) casein in PBS) for 1 hour. Saturation of the plates was analyzed alternatively by incubation with a murine monoclonal anti-hexahistidine-tag antibody or polyclonal anti-SLA rabbit serum diluted 1 : 2,000 in sample buffer (0.05% (w/v) Tween-20, 1% (w/v) casein in PBS) for 30 minutes. After washing three times, bound antibodies were detected by incubation with anti-mouse IgG HRP conjugate (Dianova, Germany) or anti-rabbit IgG HRP conjugate (Sigma-Aldrich, Germany), respectively, diluted 1 : 2,000 in sample buffer, for 30 minutes, subsequent washing as described above, followed by addition of TMB substrate (Euroimmun) for 15 minutes. Reactivities of human sera were analyzed using the same procedure, except for a different dilution of the sera (1 : 200) and the use of appropriate conjugates (Euroimmun). All incubation steps were carried out at room temperature. The OD was read at 450 nm using an automated spectrophotometer (Tecan, Germany).

#### 2.3.1. SDS-PAGE and Westernblots

Proteins were analyzed following SDS-PAGE using the NuPAGE system (Invitrogen, Germany) according to the manufacturer's instructions. In some cases, proteins were electrotransferred to nitrocellulose membranes and then used in westernblots. In the first immunological reaction human sera diluted 1 : 200, rabbit sera diluted 1 : 2,000 or a murine monoclonal antibody against hexa-histidine diluted 1 : 2.000 in universal buffer plus (Euroimmun) were applied. Bound antibodies were visualized by anti-IgG conjugated to alkaline phosphatase and NBT/BCIP (Euroimmun) as described earlier [28]. Some proteins were also analyzed by MALDI-ToF fingerprinting and MALDI-ToF tandem mass spectrometry after SDS-PAGE and tryptic cleavage [29]. Protein concentrations were determined by bicinchoninic acid assay (Sigma, Germany).

## 3. Results

### 3.1. Preparation of Recombinant SLA Proteins and a Corresponding Rabbit Serum

**Figure 1:**
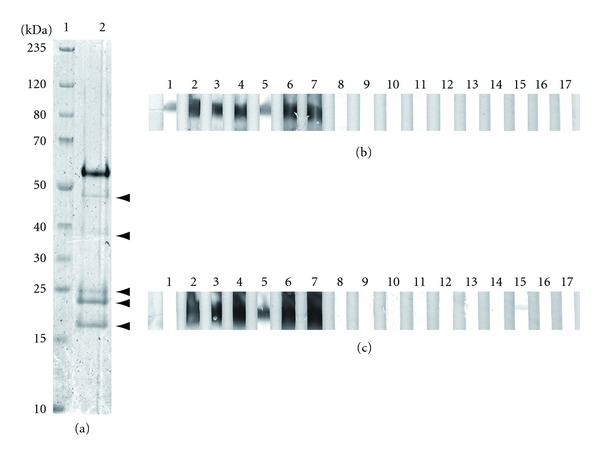
Characterization of recombinant SLA proteins by SDS-PAGE and westernblot. (a) Bacterially expressed SLA was analyzed by SDS-PAGE with coomassie staining. Lane 1: molecular mass markers, kDa indicated; lane 2: 2 *μ*g recombinant SLA, arrow heads indicate the presence of anti-His-Tag antibody reactive SLA-fragments verified by mass spectrometry, (b) 1 *μ*g/lane recombinant SLA purified from *E. coli,* (c) or cell-free supernatant from HEK293 expressing SLA; lane 1: murine monoclonal antibody against hexahistidine; lanes 2: rabbit polyclonal serum against SLA; lanes 3–7: anti-SLA positive sera from patients with autoimmune hepatitis (AIH); lanes 8–12: anti-SLA negative sera from patients with AIH; lanes 13–17: sera from healthy blood donors; the band in c, lane 15 does not correspond to SLA.

Coding DNA for human full-length SLA isoform 1 was ligated with a prokaryotic expression vector and expressed in *E. coli* (SLA-His-coli). The protein, purified by metal chelate affinity chromatography, migrated according to its calculated mass of 52 kDa when separated by SDS-PAGE (Figure 1(a)). Additionally, two minor bands corresponding to smaller masses were visible. Identities of the proteins as full-length SLA and fragments thereof were verified by MALDI-ToF fingerprinting and reactivity of a murine monoclonal antibody against hexa-histidine. Its use for the immunization of New Zealand White rabbits provoked high-titer reactivity against itself (Figure 1(b)). Anti-SLA rabbit serum reactivity in turn verified the presence of recombinant SLA in HEK293 transfected with a eukaryotic expression vector containing its coding sequence by westernblot (Figure 1(c)) and its accessibility in the indirect immunofluorescence assay. In contrast, wild-type HEK293 as well as HEK293 transfected with the same vector backbone containing an unrelated coding sequence did not produce similar results.

When SLA-expressing HEK293 were homogenized in hypotonic buffers, the recombinant protein (SLA-HEK) could be released in a soluble form as verified by presence of >80% SLA-HEK in the supernatant after ultra-centrifugation. In contrast, SLA-His-coli could only by solubilised using high concentrations of chaotropic agents.

### 3.2. Denatured SLA for Autoantibody Determination

When either SLA-His-coli or SLA-HEK were used in westernblot, all 20 sera from anti-SLA positive patients with AIH produced a band corresponding to the position of the anti-SLA rabbit serum reactivity. None of the sera from 20 anti-SLA negative AIH patients, 15 PBC patients, 15 patients with HCV infection or from 30 healthy blood donors generated bands at identical positions (Figure 1(b)).

**Figure 2:**
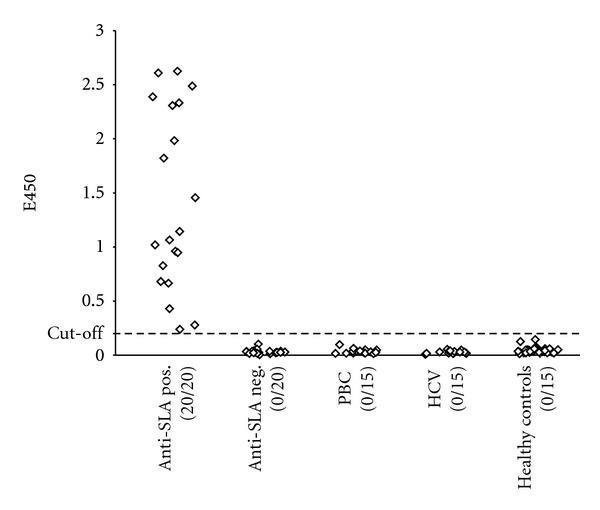
Autoantibodies against SLA detected with ELISA based on bacterially expressed protein. Bacterially expressed SLA (SLA-His-coli) was used to form the solid phase in an indirect ELISA for the determination of human IgG antibodies in 20 anti-SLA positive autoimmune hepatitis sera, 20 anti-SLA negative autoimmune hepatitis sera, 15 primary biliary cirrhosis sera, 15 HCV sera, and 30 sera from healthy blood donors. Positive and total numbers of sera are given below the diagrams. The cut-off value is presented by a dotted line.

SLA-His-coli could be immobilized to microplates and saturated the surfaces at 200 ng per well as verified by a high maximum ELISA signals (*E*_450_ > 4) and a sigmoidal saturation curve obtained after incubation of anti-hexa-histidine antibody and anti-SLA rabbit serum, respectively. Following ROC analysis, a usable cut-off value of *E*_450_ = 0.25 was defined. Similar to the westernblot, signals above the cut-off value were reached in 20 AIH sera but in none of the controls (Figure 2).

In neutralization experiments, SLA-His-coli and cytoplasmic supernatants of HEK293 expressing SLA but not similar fractions of wild-type HEK293 were able to inhibit the anti-SLA reactivities of all 20 human sera and the anti-SLA rabbit serum with SLA-His-coli in ELISA in a dose-dependent manner. Maximum inhibition rates were equal for SLA-His-coli and SLA-HEK.

### 3.3. Recombinant Cell-Based Indirect Immunofluorescence for Autoantibody Determination

**Figure 3:**
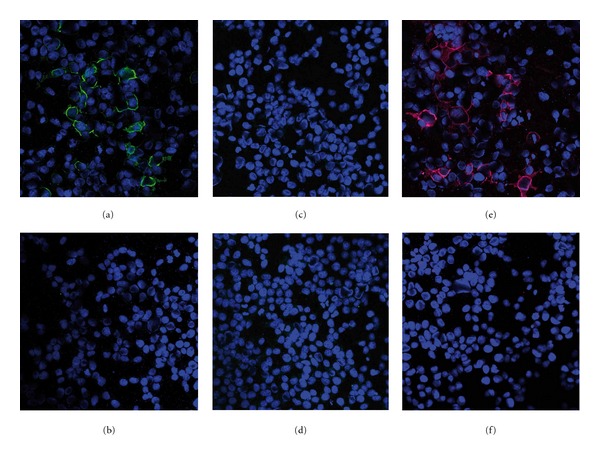
Immunofluorescence staining patterns of HEK293 expressing soluble liver antigen. HEK293 expressing soluble liver antigen (SLA) and wild-type HEK293 were incubated with either 1 : 100 diluted human serum or 1 : 100 diluted anti-SLA rabbit serum and bound antibodies were visualized with either anti-human IgG FITC (green) or anti-rabbit IgG Cy3 (red) conjugates. Nuclear DNA was stained with TO-PRO-3 (blue). (a, c, e) HEK293-SLA; (b, d, f) untransfected HEK293; (a & b) representative anti-SLA positive serum from a patient with autoimmune hepatitis; (c & d) representative anti-SLA negative serum from a healthy blood donor; (e & f) anti-SLA rabbit serum.

When HEK293 expressing SLA, either fixed with acetone or 1% (w/v) formalin in acetone, were used as substrates in indirect immunofluorescence (IFA), all 20 anti-SLA positive AIH sera produced a cytoplasmic staining pattern (Figure 3(a)) that was absent in the wild-type HEK293 (Figure 3(b)). Similar staining patterns were produced by the anti-SLA rabbit serum (Figures 3(c) and 3(d)) whereas none of the control sera reacted, independent of the fixation. Staining patterns were in all cases better defined when cells were fixed with formalin (Supplementary Figure 1 available online at  http://dx.doi.org/10.1155/2013/572815) which eased the interpretation of the results considerably, especially in the presence of antinuclear or anti-mitochondrial antibodies.

In neutralization experiments, cytoplasmic supernatants of HEK293 expressing SLA but neither similar fractions of wild-type HEK293 nor SLA-His-coli were able to abolish the reactivities of all 20 human sera with SLA-HEK in IFA in a dose-dependent manner whereas co-incubation of sera with SLA-His-coli only reduced the anti-SLA titers in the human sera but was not able to abolish the reactivities. In contrast, the reactivity of the anti-SLA rabbit serum could be neutralized by both, SLA-His-coli and SLA-HEK.

In an immunoprecipitation assay with subsequent detection of SLA by westernblot (see Supplementary Materials and Methods and Supplementary Figure 2) anti-SLA positive AIH sera precipitated SLA-HEK whereas none of the 20 anti-SLA negative AIH sera was able to pull down recognizable amounts of the eukaryotically expressed protein.

## 4. Discussion

The present study is the first to report the development of an IFA which allows the detection of anti-SLA autoantibodies, an autoantibody marker of AIH. These autoantibodies do not produce a recognizable immunofluorescent pattern by conventional indirect immunofluorescence, regardless of whether tissue or known cell line are used as antigenic substrates [5]. The reason for this is unclear but it can be speculated that either the concentration of the target antigen is too low in the respective IFA substrates to produce a staining *per se* or is significantly reduced after fixation due to its solubility, similar to the effect of easily soluble lactoferrin and myeloperoxidase after ethanol fixation of neutrophils [30, 31]. The latter view is in line with the recent identification of SLA as the *O*-phosphoseryl-tRNA(Sec) selenium transferase [7], which converts *O*-phosphoseryl-tRNA(Sec) to selenocysteinyl-tRNA(Sec). The enzyme is essential for selenoprotein biosynthesis and, as such, is primarily expressed in liver where it is located as an easily soluble protein within the cytosolic fraction [32, 33].

The recombinant HEK293 cell-based IFA (RC-IFA) is based on overexpressed SLA that is immobilized in the cytoplasmic region of the cells due the use of formalin in the fixation step. The cells show an easily visible and strong staining when incubated with anti-SLA antibodies of either human or rabbit origin (Figure 3). At the same time, the results are highly specific because non-anti-SLA reactivities can be easily distinguished from reactions with wild-type HEK293 incubated in parallel. The new anti-SLA RC-IFA will greatly assist routine laboratories which use IFA for hepatitis-related autoantibody screening. A combination of conventional IFA testing based on rodent tissue and HEp-2 and the SLA RC-IFA can detect the whole spectrum of autoantibodies that are diagnostically relevant for AIH, as they have been described by the IAIHG and the American Association for the Study of Liver Diseases (AASLD) practise guidelines [2, 12, 13]. This comprehensiveness of IFA, together with its high proficiency, may be attractive to laboratories that have previously turned away from IFA [12, 24].

Our results also indicate the presence of conformational epitopes in the HEK293-expressed SLA, confirming earlier speculations [7, 18, 21]: a cytosolic fraction of the recombinant cells was able to abolish the reactivity of human and rabbit anti-SLA antibody positive sera in RC-IFA. In contrast, the bacterially expressed homologue only showed an incomplete inhibition of human sera. On the other hand, anti-SLA antibody positive sera raised in a rabbit by immunization with SLA produced in *E. coli* could be neutralized. We speculate that this behaviour mirrors the presence of exclusively linear epitopes in the recombinant protein purified from *E. coli* inclusion bodies under denaturing conditions.

When designing the study, we expected that the developed RC-IFA would offer a much higher sensitivity than ELISA or blot assays based on prokaryotically expressed protein [14–16]. The studies done by Ma et al. [21] and Vitozzi et al. [18] had postulated that some AIH-1 and AIH-2 sera have reactivity to recombinant SLA restricted to conformational epitopes. Our results do not confirm this assumption. All sera found positive for anti-SLA by the ELISA were also positive by the RC-IFA SLA and *vice versa*. Obviously, all anti-SLA positive sera contain simultaneous reactivity to both linear and conformational epitopes. However, the AIH patient cohort was small and the study had a retrospective design based on the serological characterization of anti-SLA with established assays such that the outcome might be biased. Also, we cannot exclude the possibility that the epitopes presented by the recombinant cells are not the same with those recognised by the previously reported radioligand assays [7, 18, 21]. A large prospective study using all the available assays and well-defined sera in parallel, potentially conducted under the auspices of the IAIHG, would be needed to establish the validity of the assays.

Provided that the present findings are confirmed on larger cohorts and in other laboratories, the anti-SLA RC-IFA is able to serve as an equal supplement for ELISA or blot assays in routine laboratories and can as such be implemented in daily liver-associated autoantibody testing.

## Supplementary Material

Supplementary Figure 1: Double-staining of HEK293 expressing soluble liver antigen.
Supplementary Figure 2: Immunoprecipitation-westernblot assay using HEK293-SLA.

## Figures and Tables

**Table 1:** Primer sequences for PCR amplification of cDNA fragments of SLA. Primers were synthesized by MWG, Germany. F: forward primer; R: reverse primer.

Protein	Restriction sites	Primer sequences (5^'^–3^'^)
SLA-His-coli	BsmBI	F: ATTACGTCTCACATGAACCGCGAGAGCTTCGCGGCG
	BsmBI	R: ATTACGTCTCTTCGAGTGAAGAAGCATCCTGGTATGTGTC

SLA-HEK	BsmBI	F: ATTACGTCTCACATGAACCGCGAGAGCTTCGCGGCG
	BsmBI	R: ATTACGTCTCTTCGAGTCATGAAGAAGCATCCTGGTATGTG

## Abbreviations

AP: Alkaline phosphatase;
BSA: Bovine serum albumin;
FITC: FLuorescein isothiocyanate;
HCV: Hepatitis C virus;
HRP: Horse raddish peroxidase;
IFA: Indirect immunofluorescence assay
MALDI-ToF: Matrix-assisted laser desorption/ionization trap-time of flight mass spectrometry;
PAGE: Polyacrylamide gel electrophoresis;
RC-IFA: recombinant cell-based indirect immunofluorescence assay;
SLA: soluble liver antigen;
AASLD: American Association for the Study of Liver Diseases;
IAIHG: International AIH Group;
AIH: autoimmune hepatitis;
PBC: primary biliary cirrhosis.
