# Supplementary material for: Development of a Recombinant Cell-Based Indirect Immunofluorescence Assay for the Determination of Autoantibodies against Soluble Liver Antigen in Autoimmune Hepatitis
Source: Clin Dev Immunol. 2013 Jan 16;2013:572815. doi: 10.1155/2013/572815 (PMC3562650; doi:10.1155/2013/572815)

Supplementary Materials and Methods

Immunoprecipitation-westernblot assay

Human sera were diluted 1:200 in PBS, 0.5% (w/v) BSA, 0.2% (w/v Tween-20 sample buffer and incubated for 30 minutes with 10 µL Protein G-DynaBeads (Invitrogen, Germany) that had been equilibrated with sample buffer according to the manufacturer's instructions. Unbound antibodies were removed by three washing steps with PBS, 0.2% (w/v Tween-20. Subsequently, cell-free supernatant of HEK293 expressing SLA or non-transfected HEK293 (negative control) 1:50 diluted in sample buffer were incubated for 30 minutes followed by three washing steps. Immobilized proteins were eluted with 20 µL PBS, 5% (w/v) SDS and analyzed by westernblot.

Supplementary figure 1. Double-staining of HEK293 expressing soluble liver antigen.

HEK293 expressing soluble liver antigen (SLA) either after fixation with acetone or 1% formalin in acetone were incubated an anti-SLA positive human serum (1:100) and anti-SLA rabbit serum (1:80) and bound antibodies were visualized with anti-human IgG FITC (green) or anti-rabbit IgG Cy3 (red) conjugates. Nuclear DNA was stained with TOPRO3 (blue). (A-D) acetone-fixed cells; (E-H) formalin/acetone-fixed cells; (A-C & D-G) individual fluorescence patterns; (D & H) merged pictures.

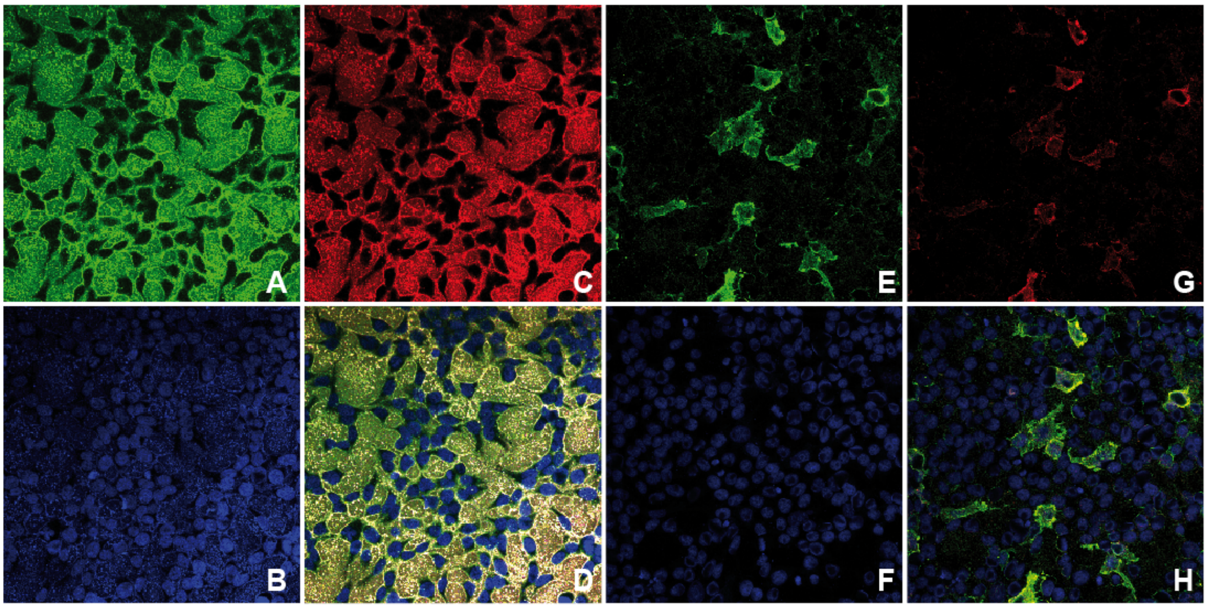

Supplementary figure 2. Immunoprecipitation-westernblot assay using HEK293-SLA.

Immunoglobulin G from human sera was immobilized on protein G-coated magnetic beads and subsequently used to pull-down antigens from cell-free supernatants of HEK293 expressing SLA and wild-type HEK293, respectively. SLA was detected by westernblot using the anti-SLA rabbit serum (1:2,000). rc, HEK293 expressing SLA; wt, wild-type HEK293.

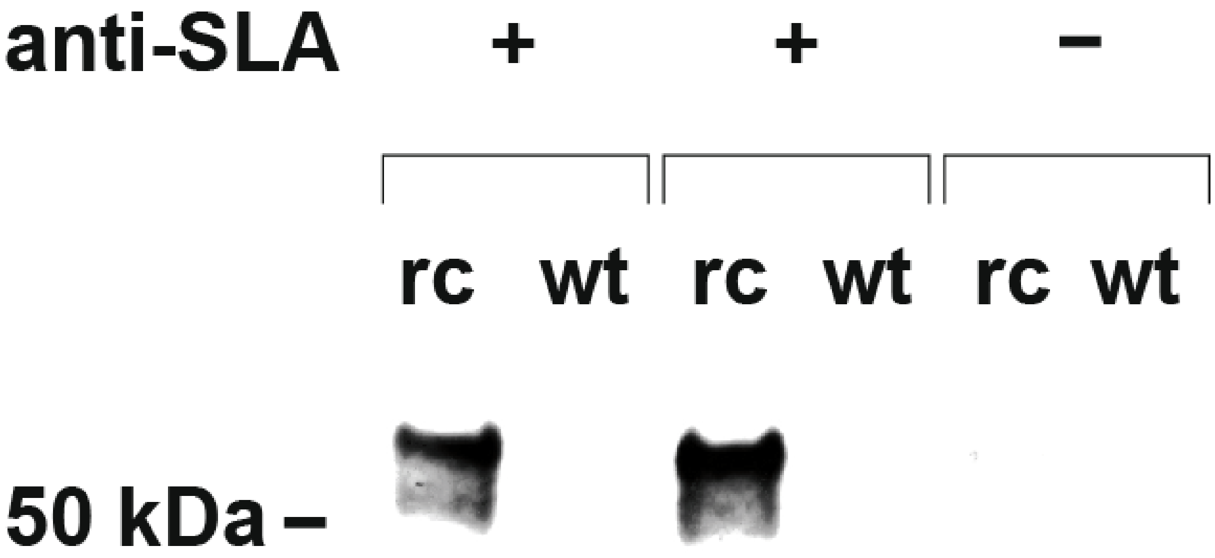

Supplement: Supplementary file 1 — Supplementary Figure 1: Double-staining of HEK293 expressing soluble liver antigen. Supplementary Figure 2: Immunoprecipitation-westernblot assay using HEK293-SLA. [file 572815.f1.pdf]
